# 不同方法和标本检测华氏巨球蛋白血症患者MYD88突变的初步探索

**DOI:** 10.3760/cma.j.issn.0253-2727.2022.05.007

**Published:** 2022-05

**Authors:** 怡 陶, 增凯 潘, 硕 王, 黎 王, 维莅 赵

**Affiliations:** 上海血液学研究所，医学基因组学国家重点实验室，国家转化医学中心（上海），上海交通大学医学院附属瑞金医院，上海 200025 Shanghai Institute of Hematology, State Key Laboratory of Medical Genomics, National Research Center for Translational Medicine at Shanghai, Ruijin Hospital Affiliated to Shanghai Jiao Tong University School of Medicine，Shanghai 200025, China

**Keywords:** Waldenstrom巨球蛋白血症, 髓样分化因子88, 二代测序, 聚合酶链反应, Waldenstrom Macroglobulinemia, Myeloid differentiation factor 88, Next-generation sequencing, Polymerase chain reaction

## Abstract

**目的:**

探索如何提高华氏巨球蛋白血症（WM）患者MYD88突变检测的阳性率和准确率。

**方法:**

回顾性分析2017年6月至2021年6月上海交通大学医学院附属瑞金医院66例初诊WM患者MYD88突变检测结果，分析不同方法和标本检测MYD88突变的阳性率和准确率。

**结果:**

66例WM患者中51例MYD88突变阳性，整体阳性率77％。根据检测方法分类：二代测序（NGS）和等位基因特异性PCR（AS-PCR）检测突变阳性率显著高于一代测序Sanger法（84％对71％对46％，*P*<0.05）。根据标本取材部位分类：淋巴结和骨髓标本检测突变阳性率显著高于外周血标本（79％对84％对52％，*P*<0.05）。NGS检测淋巴结、骨髓和外周血MYD88突变阳性率分别为86％、90％和67％。AS-PCR检测淋巴结、骨髓和外周血MYD88突变阳性率分别为78％、81％和53％。39例WM患者进行≥2次MYD88突变检测，以每例患者最终突变检测结果作为标准，判断不同方法和标本的准确率。淋巴结（18例）和骨髓（13例）NGS检测准确率显著高于外周血（4例）标本（100％对100％对75％，*P*<0.05）。淋巴结（15例）、骨髓（11例）和外周血（16例）AS-PCR检测准确率的差异无统计学意义（93％对91％对88％，*P*>0.05）。

**结论:**

在检测WM患者MYD88突变情况方面，NGS或AS-PCR法优于Sanger法，淋巴结和骨髓标本优于外周血标本。

## Abstract (en)

**Objective:**

To improve the positivity rate and accuracy of MYD88 mutation detection in patients with Waldenström macroglobulinemia（WM）.

**Methods:**

MYD88 mutation status was retrospectively evaluated in 66 patients diagnosed with WM in Ruijin Hospital Affiliated to Shanghai Jiao Tong University School of Medicine from June 2017 to June 2021. The positivity rate and accuracy of the different methods and specimens for MYD88 mutation detection were analyzed.

**Results:**

MYD88 mutations were detected in 51 of 66 patients with WM, with an overall positivity rate of 77％. The positivity rate of the next-generation sequencing（NGS）or allele-specific polymerase chain reaction（AS-PCR）was significantly higher than that of the first-generation Sanger sequencing（84％ *vs* 71％ *vs* 46％, *P*<0.05）. For the different specimens, the positivity rate for the lymph nodes or bone marrow was significantly higher than that of peripheral blood（79％ *vs* 84％ *vs* 52％, *P*<0.05）. The positivity rate of the MYD88 mutation in the lymph nodes, bone marrow, and peripheral blood determined by NGS was 86％, 90％, and 67％, respectively. The positivity rate in the lymph nodes, bone marrow, and peripheral blood detected by AS-PCR was 78％, 81％, and 53％, respectively. Thirty-nine patients with WM underwent ≥ 2 MYD88 mutation detections. The final MYD88 mutational status for each patient was used as the standard to determine the accuracy of the different methods and in different specimens. The accuracy of MYD88 mutation detection in the lymph nodes（*n*＝18）and bone marrow（*n*＝13）by NGS was significantly higher than that in the peripheral blood（*n*＝4）（100％ *vs* 100％ *vs* 75％, *P*<0.05）. There was no statistically significant difference in the accuracy of MYD88 mutation detection by AS-PCR in the lymph nodes（*n*＝15）, bone marrow（*n*＝11）, or peripheral blood（*n*＝16）（93％ *vs* 91％ *vs* 88％, *P*>0.05）.

**Conclusion:**

In the detection of the MYD88 mutation in patients diagnosed with WM, NGS or AS-PCR is more sensitive than Sanger sequencing. Lymph nodes and bone marrow specimens are better than peripheral blood specimens.

华氏巨球蛋白血症（WM）是一种分泌单克隆IgM的惰性淋巴浆细胞淋巴瘤，在非霍奇金淋巴瘤中占2％[1]。WM病程长，疾病易发生进展，影响患者的生活质量和预后。国外文献报道，90％的WM患者伴MYD88L265P突变，国内关于WM患者MYD88突变检测的报道较少，且各中心报道的突变率不一致[2]。Treon等[3]报道，布鲁顿酪氨酸激酶抑制剂（BTKi）单药的疗效与MYD88及CXCR4突变状态有关。MYD88^MUT^CXCR4^WT^患者预后最佳，其次是MYD88^MUT^CXCR4^MUT^，MYD88^WT^CXCR4^WT^患者预后最差。基于MYD88突变在WM诊断和BTKi疗效判断中的价值，本中心通过不同方法和标本检测66例WM患者MYD88突变情况（部分患者通过不同方法和标本进行多次检测），初步探索如何提高MYD88突变检测阳性率和准确率。

## 病例与方法

1. 一般资料：回顾性分析2017年6月至2021年6月上海交通大学医学院附属瑞金医院进行MYD88突变检测的66例初诊WM患者。所有患者符合以下WM诊断标准：①血清中存在单克隆IgM；②病理检查证实骨髓中存在淋巴浆细胞浸润；③除外其他已知类型的淋巴瘤[4]–[5]。

2. MYD88突变检测标本和方法：66例WM患者进行113例次MYD88突变检测，检测方法包括一代测序（Sanger法）、等位基因特异性聚合酶链反应（AS-PCR）和二代测序（NGS），标本来源包括淋巴结、骨髓和外周血。

3. 外周血、骨髓和淋巴结肿瘤组织基因组DNA通过DNA纯化试剂盒提取并定量。外周血和骨髓未经CD19分选。留取标本均获得患者的知情同意并签署知情同意书。

4. NGS检测平台和深度：将基因组DNA片段化，构建DNA文库（200～250 bp）。用探针靶向捕获技术富集目的DNA片段，按照 Illumina成对末端测序文库试剂盒说明书，使用 150 bp成对末端reads构建上机文库。以定量试剂盒进行定量后，应用 Illumina Novaseq平台对MYD88基因进行测序。肿瘤DNA样本的测序深度为1500～5000 ×。

5. 统计学处理：采用 SPSS 23.0 软件进行统计学分析。组间率的比较采用似然比*χ*^2^检验，*P*<0.05为差异有统计学意义。

## 结果

1. MYD88突变检测整体情况：66例WM患者中，51例患者检测出MYD88突变阳性，MYD88突变阳性率为77％，包括50例MYD88L265P突变，1例MYD88L273P突变。其中39例患者初诊时进行不同方法和标本多次检测，因此MYD88突变检测共113例次。

2. 不同方法检测MYD88突变：113例次MYD88突变检测中，检测方法以NGS和AS-PCR为主，其中NGS方法49例次（43％），AS-PCR方法51例次（45％），Sanger法13例次（12％）。NGS方法检测MYD88突变阳性率为84％，AS-PCR方法阳性率为71％，而Sanger法阳性率为46％，NGS和AS-PCR检测MYD88突变阳性率均显著高于Sanger法（*P*值均<0.05）。

3. 不同标本检测MYD88突变：113例次的MYD88突变检测中，检测标本以淋巴结和骨髓为主，其中淋巴结标本47例次（41％），骨髓标本37例次（33％），外周血29例次（26％）。淋巴结标本MYD88突变检测阳性率为79％，骨髓阳性率为84％，外周血阳性率为52％，淋巴结和骨髓标本检测MYD88突变阳性率均显著高于外周血标本（*P*值均<0.05）。

4. 相同方法检测不同标本MYD88突变：49例采用NGS检测的WM患者中，淋巴结标本MYD88突变阳性率为86％（21例），骨髓MYD88突变阳性率为90％（19例），外周血MYD88突变阳性率为67％（9例），三种标本的差异无统计学意义（*P*值均>0.05）。51例采用AS-PCR检测的WM患者中，淋巴结标本MYD88突变阳性率为78％（18例），骨髓MYD88突变阳性率为81％（16例），外周血MYD88突变阳性率为53％（17例），骨髓MYD88突变阳性率显著高于外周血（*P*＝0.040）。

5. 不同方法和标本多次检测MYD88突变：66例WM患者中，39例患者进行了≥2次的MYD88突变检测（图1）。某一特定方法+标本组合检测MYD88突变准确次数（与图1第1行该例患者最终MYD88突变结果比较）/该组合总检测次数＝该组合准确率。结果表明，NGS检测淋巴结（18例）和骨髓（13例）的准确率达100％，显著高于NGS检测外周血（4例）标本的准确率75％（*P*＝0.030）。通过AS-PCR检测淋巴结的准确率为93％（15例），骨髓的准确率为91％（11例），外周血准确率为88％（16例），三者的差异无统计学意义（*P*>0.05）。在同一WM患者中比较不同检测组合检测MYD88突变的结果，更能直观反映不同组合的准确性和敏感性。例13通过NGS检测淋巴结显示MYD88突变阳性，而NGS检测外周血显示MYD88突变阴性；例22通过AS-PCR检测淋巴结显示MYD88突变阳性，而AS-PCR检测外周血显示MYD88突变阴性；例38通过Sanger法检测淋巴结显示MYD88突变阳性，而Sanger法检测外周血显示MYD88突变阴性；例39通过Sanger法检测骨髓显示MYD88突变阳性，而Sanger法检测外周血显示MYD88突变阴性。以上4例患者通过自身对照证实，无论使用何种检测方法，骨髓或淋巴结标本均优于外周血。例33通过NGS检测骨髓显示MYD88突变阳性，而AS-PCR检测骨髓显示MYD88突变阴性；例39通过NGS检测外周血显示MYD88突变阳性，而Sanger法检测外周血显示MYD88突变阴性。提示对于相同患者、相同标本，NGS方法可能更为敏感。此外，例13通过AS-PCR检测骨髓显示MYD88突变阳性，而NGS检测外周血显示MYD88突变阴性，提示尽管NGS敏感性高，仍不能克服外周血标本的假阴性。例19通过NGS检测骨髓显示MYD88突变阳性，而PCR检测淋巴结显示MYD88突变阴性，提示通过NGS检测骨髓标本可能是较优选择。

**图1:**
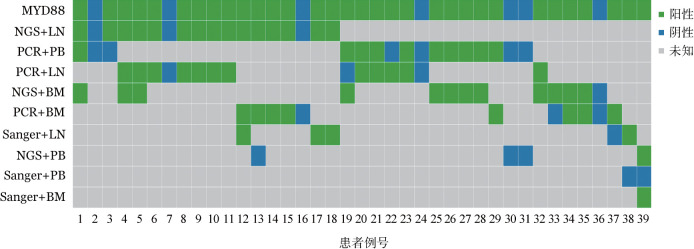
不同方法和标本检测39例华氏巨球蛋白血症（WM）患者的MYD88突变情况 LN：淋巴结；BM：骨髓；PB：外周血；第一行为患者MYD88突变的最终结果（WM患者进行多次检测时，1次MYD88突变阳性即认为该患者阳性；若多次检测均阴性，则认为该例患者阴性）

## 讨论

基于MYD88突变在WM诊断、BTKi治疗及预后方面的重要价值，本中心对66例WM患者进行多种方法和多个标本的MYD88突变检测，总体MYD88突变阳性率为77％，其中1例患者为非MYD88L265P突变（MYD88L273P突变）。

Sanger法曾在MYD88突变检测中广泛应用，也能检测非L265P突变，但敏感性较低，不能发现<20％的突变细胞。如果DNA来源的标本未经富集，假阴性率可高达30％～50％[6]。Gachard等[7]报道，Sanger法检测未经分选骨髓的MYD88突变阳性率为67％。与此一致，本中心Sanger法总体阳性率仅46％，显著低于AS-PCR和NGS（*P*<0.05）。观察同一患者的系列结果，我们发现例37、例38和例39均出现Sanger假阴性结果，也再次证实Sanger法的不足。对于标本的选择，我们观察到淋巴结和骨髓优于外周血（*P*<0.05），应用Sanger法检测外周血标本时假阴性率更高（例38和例39）。敏感性较低的Sanger法比较适合CD19磁珠富集外周血，或在富集条件不允许的情况下采用淋巴结或骨髓标本，以尽可能降低检测的假阴性率。

针对L265P点突变的AS-PCR法敏感性较高，达1％[8]，且检测成本可控，缺点是只能检测L265P突变。国外报道显示，AS-PCR检测CD19分选骨髓的MYD88突变阳性率达到93％～97％[9]–[10]，而国内类似方法检测阳性率为73％～94％[11]–[12]。本中心AS-PCR方法检测MYD88突变阳性率为71％，可能与包含较多外周血标本（17例）有关。Xu等[13]报道，AS-PCR检测未分选外周血的阳性率为39.5％。与之类似，本中心AS-PCR检测外周血阳性率为53％，显著低于AS-PCR检测骨髓标本阳性率81％（*P*<0.05）。上述结果提示，即使AS-PCR敏感性较高，也无法克服外周血较高的假阴性率，而例22也补充证实AS-PCR检测外周血可能出现假阴性。

NGS的敏感性高，Nakamura等[14]报道其敏感性达0.02％，较AS-PCR高5倍。其次，NGS能检测非L265P突变并兼顾WM中40多种CXCR4突变类型[15]。NGS可以定量监测MYD88突变频率，而突变频率能间接反映WM的肿瘤负荷[16]。本中心NGS法检测MYD88突变阳性率最高，为84％。从标本选择来看，NGS检测骨髓阳性率最高，为90％，NGS检测外周血阳性率最低，为68％。分析结果表明，NGS检测骨髓或淋巴结的准确率均高于外周血（*P*<0.05），且例13也证实NGS检测外周血也可能出现假阴性。本中心WM患者整体MYD88突变阳性率为77％，低于国外文献报道，原因可能是25％的检测标本是未经CD19分选的外周血，12％的标本采用Sanger法检测。而根据本中心39例WM患者不同检测组合的MYD88突变结果，外周血和Sanger法均存在假阴性情况。

标本进行CD19富集能提高MYD88检测阳性率，特别是针对不敏感的Sanger法和肿瘤负荷较低的标本如外周血。对于骨髓标本是否一定需要CD19分选，仍有不同意见。WM患者初诊时通常有单克隆B细胞和单克隆浆细胞两群WM的重要组成细胞[17]，Gustine等[18]报道AS-PCR检测经治WM患者CD19分选后的骨髓标本，有6例患者MYD88突变阴性，而采用同样方法检测这6例患者未分选的骨髓标本却显示MYD88突变阳性。Gustine等[18]推测其原因可能是治疗减少了B细胞克隆，但残存的浆细胞克隆对CD19分选不敏感，造成假阴性，而未分选的骨髓标本不受影响。对于NGS是否一定最敏感，也存在争议。Kofides等[19]发现NGS检测WM患者骨髓MYD88突变的阳性率为66％，而同样的标本通过AS-PCR检测阳性率达96％，因此作者得出结论NGS不能敏感检测MYD88突变。我们发现此研究中尽管NGS方法平均测序深度为1 500 ×（范围305～3 707 ×），但敏感性仅5％，甚至低于AS-PCR方法。而本中心NGS方法测序深度较高，且测序前对标本目的片段行PCR扩增，因此NGS敏感性可达0.09％。更准确的结论有待未来行多中心、大样本研究证实。

本中心的初步研究提示，靶向NGS检测骨髓或淋巴结标本能敏感检出MYD88（包括L265P和非L265P）突变；若无法行NGS，推荐应用AS-PCR检测骨髓MYD88突变。检测标本方面，推荐初诊患者标本经CD19分选后行NGS/AS-PCR检测，尽量避免外周血标本；而对于CD20单抗治疗后的WM患者，未经分选的骨髓标本可能更佳。

目前WM患者疗效评价以IgM水平为标准[20]，但文献报道IgM水平和临床参数有不一致情况[3]。Xu等[13]报道，定量AS-PCR显示WM经治患者较初诊患者等位基因突变频率降低，ΔCt值升高，提示定量监测MYD88突变频率可能成为疗效判断的替代参数。此外，Wu等[21]采用定量AS-PCR检测WM患者外周血细胞游离DNA（cfDNA）的阳性率为85.2％，敏感性为0.4％，与AS-PCR检测骨髓标本一致性达96.3％。因此，外周血cfDNA检测MYD88突变敏感且创伤较小，值得推广。WM患者MYD88突变检测仍有较大的改善空间和临床应用前景，值得未来进一步探索。
